# Historical Ethnobotany: Interpreting the Old Records

**DOI:** 10.3390/plants12213673

**Published:** 2023-10-25

**Authors:** Raivo Kalle, Renata Sõukand

**Affiliations:** 1 Estonian Literary Museum, Vanemuise 42, 51003 Tartu, Estonia; 2 Department of Environmental Sciences, Informatics and Statistics, Ca’ Foscari University of Venice, Via Torino 155, Mestre, 30172 Venice, Italy; renata.soukand@unive.it

For centuries, knowledge about the use of plants has been collected, published, or simply left in archives. Today, however, we live in a world with an abundance of information, and modern scientific standards put pressure on us, as researchers, to collect more and more data continuously. In current ethnobotany, publishing research with new data collected during fieldwork is much faster and easier. Quiet time for the qualitative analysis of previously collected data may seem unimportant; however, this Special Issue aims to emphasize that authors should utilize previously collected or stored ethnobotanical information in their research and, as such, this practice could be considered pioneering. This practice would also aid in paying tribute to our colleagues who came before us and collected and valued these data. In addition, this knowledge is essential in understanding changes in the environment and plant use as well as attitudes towards plants more broadly. It is encouraging that so many research articles focus on the cultural significance of a particular plant. In the case of medicinal plants, a growing number of scholars have begun to understand that their cultural, historical, and religious significance is also important, in addition to the active substances obtained from these plants. This Special Issue also covers the topics of ethnoveterinary medicine and dyeing with plants, which are rarely addressed in European ethnobotany. Furthermore, this collection includes more unusual approaches to studying the use of plants, for example, their representation in early folk songs and paintings. Moreover, it is indeed a pleasure that colleagues who have not dealt with and/or studied ethnobotany before have contributed to this collection.

We arranged the current introduction on a diachronic scale, starting with those who utilized the earliest available sources.

Edelman et al. [1] and Dal Cero et al. [2] mapped the most distant past and presented knowledge that is up to 2000 years old. In their comprehensive review, Edelman et al. [1] detailed the use of small freshwater plants, known as duckweeds (Lemnaceae Martinov), from ancient times to the Middle Ages. In addition, they compared the uses of this group of plants in different civilizations—Chinese, Christian, Greek, Hebrew, Hindu, Japanese, Mayan, Muslim, and Roman. They found that the use of duckweeds was already geographically widespread in antiquity and that they were integrated into classical cultures in the Americas, Europe, the Middle East, and the Far East. Dal Cero et al. [2] reviewed medicinal plant use in Central Europe from the earliest available records. They found that of the same 102 medicinal plants circulating in herbals from ancient times, more than half have retained similar uses regardless of the changes in both medicine and technology that have taken place since this period. The value of their work is that they confirm the concept of the social validation of plant uses. Thus, traditional and long-standing medicinal plants form the basis for regulatory sources of traditional herbal use.

De Vahl et al. [3] studied the period that began with the Middle Ages, revealing that the first reports of medicinal uses of *Peucedanum ostruthium* (L.) Koch (Apiaceae), naturally occurring in the mountainous regions of central and southern Europe, date back to the 13th century in the Nordic countries. This species was first cultivated in Sweden in the 17th–19th centuries and was known as the primary drug used in ethnoveterinary medicine. Today, this species has been preserved in specific locations in Sweden, in former cultural areas, and has become a part of the country’s biocultural heritage. The authors emphasize that during the reconstruction of former farm gardens in open-air museums, the culturally important species of the past should also be highlighted. Pinke et al. [4] examined the time period from the late Middle Ages (1578) to the present day. They reviewed the folk uses and cultural significance of three field weeds in Hungary—*Papaver rhoeas*, *Centaurea cyanus*, and *Delphinium consolida*. They found that these species were used for medicinal and ornamental purposes, in religious celebrations, and in children’s games during the time period studied. In addition, they found that they play an essential role in folk art and folk poetry. The height of the cultural importance of field weeds was in the early 20th century. Since the decline of field weeds, beginning at the end of the 20th century, their cultural importance has drastically decreased. The general conclusion of the authors is that in addition to preserving the natural species diversity of fields, it is also necessary to consider the cultural importance of plants.

Jasprica et al. [5] studied how plants were depicted in Baroque art on the eastern coast of the Adriatic Sea in the modern era. It must be mentioned that their approach is quite innovative in the field of ethnobotany. They found that 23 different plant species were portrayed in art at the time, with 71% of them considered “exotic” species. The exotic species came from the Palearctic region (Eurasia) and the American continent. *Lilium candidum*, *Acanthus mollis*, and *Chrysanthemum* cf. *morifolium* were the most represented taxa in the paintings. The researchers believe that the plants represented in the art were chosen for their decorative appearance and symbolic importance. Milani et al. [6] travel back in time with their research to 18th century Europe and conclude their research in the present day. They compared the earliest manuscript record from Valle Imagna (Bergamo, Italy) with later sources and data collected in the area during the present time period. The value of this research is that the authors worked through a vast amount of literature. Their study revealed only a few overlaps between current and 18th century plant use—only 34 species overlapped out of the 200 species mentioned in the manuscript. The most significant change occurred in this valley in the 1960s–1970s when most of the population emigrated from the region. However, the general use of medicinal plants is fading, as the use of only 42 species was identified in recent fieldwork.

Altogether, five studies focus on the 19th century. Prakofjewa et al. [7] analyzed the folk use of medicinal plants from three early sources. The examined sources were all published in the territory of today’s Baltic states in 1829, 1891, and 1895, and a total of 219 species were identified in these reports. The authors also found that although the three early sources describe plant uses that overlap geographically, they were still quite different, with only 14 species overlapping in all three sources and 27 others mentioned in two of the sources. This indicates high biocultural diversity and dependence on local plant taxa in the past. Comparing these data with the book published by the Greek physician Pedanius Dioscorides (AD 40–90) revealed that as many as 46% of the plants mentioned by two or three authors overlapped. Overall, the presence of plants mentioned by Dioscorides was 26%. Köhler et al. [8] analyzed available information on plants used for dyeing in an 1883 questionnaire. It was found that 74 species are used in present-day Poland, Ukraine, and Belarus, the most popular of which was the onion. The authors state that most of the plants mentioned were widely known dyeing plants. However, plant dyeing is practically forgotten in Poland today, and this article may contribute to the re-emerging tradition of plant dyeing in the region.

Kalle et al. [9] looked at the period of 1891 to 1893. Dr Mihkel Ostrov carried out one of the first collections solely focused on ethnopharmacology, using national newspapers to distribute appeals for data collection to the population at large. With this action, Ostrov can be considered as one of the first individuals to use citizen science in ethnomedical data collection. In addition to appeals, Ostrov gave his correspondents feedback through said newspapers and provided motivation to the population to continue collecting such data. Using such a method, Ostrov obtained one of the highest quality collections of his time. Sõukand and Kalle [10] based their study on reports on herbal medicine, collected from three parishes bordering Russia between 1888 and 1996, which are stored in archives. In total, one hundred and nineteen species were identified. The authors observed a great variety of plant names and significant plant heterogeneity, especially in the earlier archival sources. Archival sources also provide a good context for understanding the use of medicinal plants in the past. The authors also emphasize that appropriate research methods must be used when identifying plants in archival sources because mistakes can easily occur in plant identification in historical sources. Fišer [11] focused on a specific analysis of plant lore in the folk songs examined by ethnologist Karel Štrekelj between 1895 and 1912. Plants were mentioned in 14% of the songs of the time. Among the 93 species mentioned, there were a surprising number of cultivated and exotic species, while only 42% were local wild taxa. Therefore, folk songs are also important for evaluating the relationship between man and nature.

Mattalia et al. [12] took the 20th century as the basis of their research, comparing their data collected in the Trentino–South Tyrol region of northern Italy (in 2022) with data previously gathered in 1989. While 75% of the species overlapped in both studies, the introduction of “new” plants has already occurred through the media. They also point out how courses teach people about local plant use in the region and that such bottom-up initiatives should be encouraged more. However, comparing the two regions showed that medicinal plants were used more in the border region (South Tyrol). Pieroni et al. [13] examined, through a case study of the Maronite community residing in the small village of Kormakitis, Northern Cyprus, how people who have lived in the same cultural space in the Mediterranean region for centuries have adapted their plant use. For many centuries, the Maronite minority living in the area have adapted their wild vegetable foraging to the Greek majority through long-standing cultural exchange. However, what was documented was mainly the memory of historical use, while currently, wild vegetables are foraged by a very limited number of people.

## Data Availability

Not applicable.
